# A New Analytic Formula for Minority Carrier Decay Length Extraction from Scanning Photocurrent Profiles in Ohmic-Contact Nanowire Devices

**DOI:** 10.1038/s41598-019-46020-2

**Published:** 2019-07-01

**Authors:** Cheng-Hao Chu, Ming-Hua Mao, Che-Wei Yang, Hao-Hsiung Lin

**Affiliations:** 1 Graduate Institute of Electronics Engineering, National Taiwan University, No. 1, Roosevelt Rd. Sec. 4, Taipei, 10617 Taiwan; 2 Department of Electrical Engineering, National Taiwan University, No. 1, Roosevelt Rd. Sec. 4, Taipei, 10617 Taiwan; 3 Graduate Institute of Photonics and Optoelectronics, National Taiwan University, No. 1, Roosevelt Rd. Sec. 4, Taipei, 10617 Taiwan

**Keywords:** Optical techniques, Optical physics

## Abstract

Spatially resolved current measurements such as scanning photocurrent microscopy (SPCM) have been extensively applied to investigate carrier transport properties in semiconductor nanowires. A traditional simple-exponential-decay formula based on the assumption of carrier diffusion dominance in the scanning photocurrent profiles can be applied for carrier diffusion length extraction using SPCM in Schottky-contact-based or p-n junction-based devices where large built-in electric fields exist. However, it is also important to study the electric-field dependent transport properties in widely used ohmic-contact nanowire devices where the assumption of carrier diffusion dominance is invalid. Here we derive an analytic formula for scanning photocurrent profiles in such ohmic-contact nanowire devices under uniform applied electric fields and weak optical excitation. Under these operation conditions and the influence of photo-carrier-induced electric field, the scanning photocurrent profile and the carrier spatial distribution strikingly do not share the same functional form. Instead, a surprising new analytic relation between the scanning photocurrent profile and the minority carrier decay length was established. Then the derived analytic formula was validated numerically and experimentally. This analytic formula provides a new fitting method for SPCM profiles to correctly determine the minority carrier decay length, which allows us to quantitatively evaluate the performance of nanowire-based devices.

## Introduction

Spatially resolved current measurements such as scanning photocurrent microscopy (SPCM)^1–6^ and the electron beam induced current (EBIC) technique^7–9^ have been intensively applied to explore carrier transport properties in semiconductor nanowires directly. These techniques provide valuable information for a wide range of nanowire-based devices including photodetectors^10–13^, sensors^14^, lasers^15,16^, and transistors^17–22^. Numerical studies on SPCM were also reported^23,24^. Carrier diffusion length extraction using SPCM or EBIC can be carried out in Schottky-contact-based^6,24^ or p-n junction-based devices^4,7^ with a simple-exponential-decay formula based on the assumption of carrier diffusion dominance in determining the scanning photocurrent profiles under normal excitation conditions. On the other hand, it is also important to study the electric-field dependent transport properties in widely used ohmic-contact nanowire devices^10,12,13,17,18,20–22^. Since there is no built-in electric field in ohmic-contact devices, the photo-carrier-induced electric field will make a non-negligible contribution to photocurrent, and the assumption of carrier diffusion dominance is no longer valid in such two-terminal nanowire devices with ohmic contact on both sides. Here in this study, a new analytic formula was derived from the theoretical model including the effect of the photo-carrier-induced electric field in order to extract the important parameter- carrier decay lengths from the scanning photocurrent profiles in ohmic-contact nanowire devices. Numerical simulations in InAs and Si nanowires were applied for the model verification. The effectiveness of the analytic formula in nanowires with different mobility values under different pumping and bias conditions was thoroughly discussed. In order to study the electric-field-dependent drift mechanism in nanowires experimentally, two-terminal devices with ohmic contact on both sides will be used in SPCM experiments. Due to the superior transport properties and easy fabrication of ohmic contact^25^, InAs is a suitable candidate for such measurements. From the measurements performed on InAs nanowires, electric-field dependence of the carrier decay length was directly observed, from which the mobility-lifetime product and the carrier diffusion length can be obtained. Therefore, our analytic formula was experimentally confirmed. This analytic formula can be applied in SPCM as well as EBIC measurements.

## Results and Discussion

**Figure 1:**
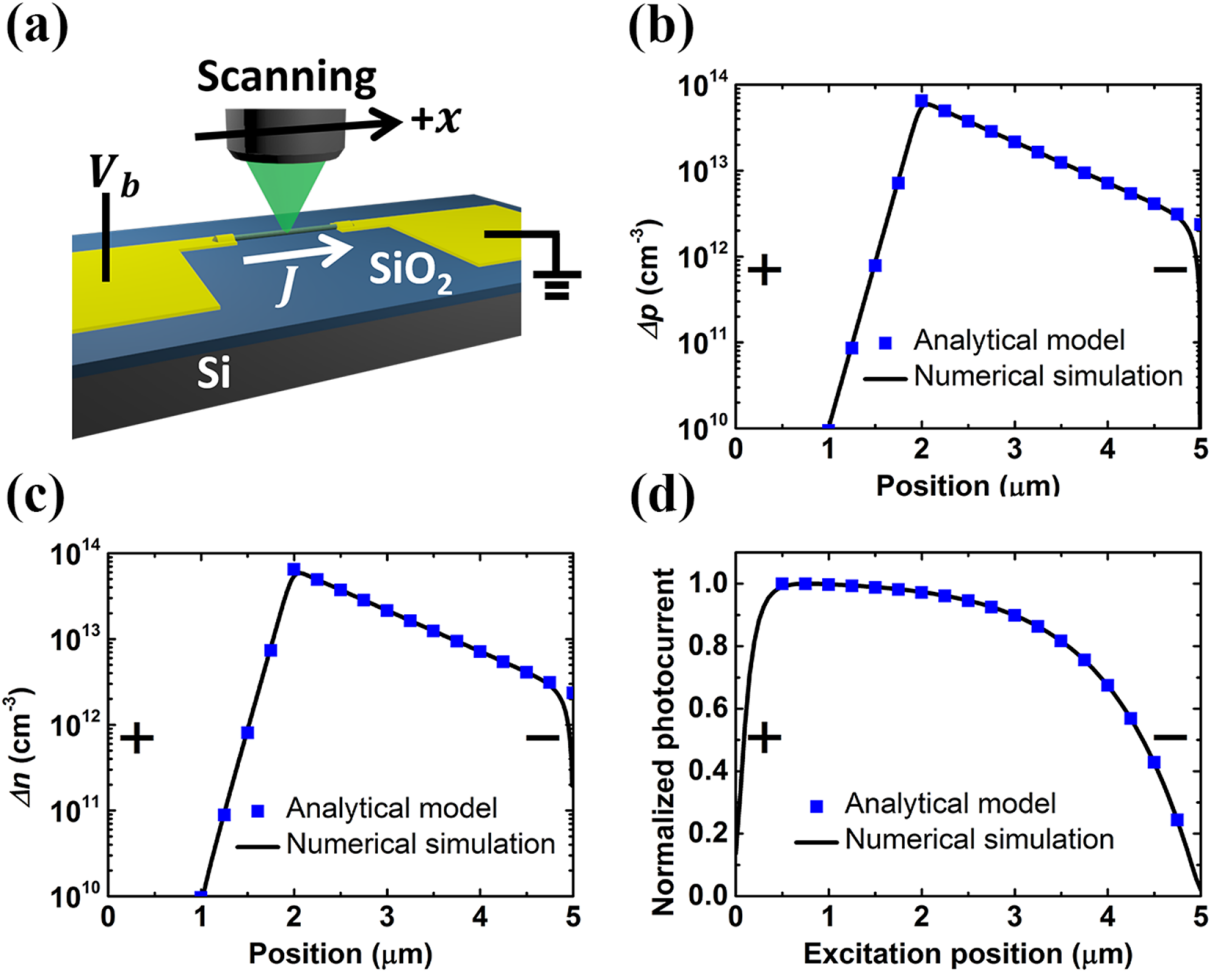
(**a**) Schematic of a typical SPCM setup. (**b**,**c**) Calculated spatial distribution of photo-induced (**b**) hole concentration and (**c**) electron concentration for n-type InAs nanowires with bias voltage *V*_*b*_ of 1 V (corresponding to an applied electric field of 2000 V/cm) under excitation at position of 2 μm. (**d**) Calculated scanning photocurrent profiles. Results from numerical simulation are also shown for comparison.

First of all, an analytic formula for scanning photocurrent profiles under electrical bias and weak optical excitation will be derived in the following. Typically, Poisson’s equation and two continuity equations are applied to describe the carrier transport in semiconductors^26,27^. Consider one-dimensional transport in a two-terminal ohmic-contact nanowire device as shown in Fig. 1a. This device has the anode at *x* = 0 and the cathode at *x* = *L*_*ch*_, where *L*_*ch*_ is the nanowire length between electrodes. We may write

$$\begin{array}{c}\frac{q}{\varepsilon }(p-n+{N}_{D}-{N}_{A})=\frac{\partial E}{\partial x}\\ \frac{\partial n}{\partial t}=\frac{1}{q}\frac{\partial {J}_{n}}{\partial x}+G-R=0,\,{J}_{n}=q{\mu }_{n}En+q{D}_{n}\frac{\partial n}{\partial x}\\ \frac{\partial p}{\partial t}=-\,\frac{1}{q}\frac{\partial {J}_{p}}{\partial x}+G-R=0,\,{J}_{p}=q{\mu }_{p}Ep-q{D}_{p}\frac{\partial p}{\partial x}\end{array}$$

where *n* and *p* are the electron and hole concentrations. *N*_*D*_ and *N*_*A*_ are the ionized donor and acceptor concentrations, respectively. Uniform dopant distribution is assumed in this study. *E* is the electric field, *q* is the fundamental charge, and *R* is the net recombination rate. *J*_*n*_ and *J*_*p*_ are the electron and hole current densities, respectively. *μ*_*n*,*p*_ and *D*_*n*,*p*_ are the electron/hole mobilities and diffusion coefficients of the nanowire, respectively. For carrier transport in the steady state, the time derivatives of the electron and hole concentrations are both zero. The generation rate *G* by optical excitation in this model is assumed to be a delta function centered at *x*_*pump*_. Later we should see that the analytical model predicts scanning photocurrent profiles which are consistent with simulation results by optical excitation of finite spot size.

When the nanowire is optically pumped, the electron concentration, the hole concentration, and the electric field differ from their values without excitation. These variables can be expressed as their values without excitation plus the optical-excitation-induced changes, i.e. *n* = *n*_0_ + Δ*n*, *p* = *p*_0_ + Δ*p*, and *E* = *E*_0_ + Δ*E*, where *n*_0_, *p*_0_, and *E*_0_ are the values without excitation and Δ*n*, Δ*p*, and Δ*E* are the optical-excitation-induced changes. An n-type semiconductor nanowire is taken here as an example. Thus, *N*_*D*_ ≫ *N*_*A*_ is assumed in Poisson’s equation. The continuity equation for minority carriers can be expressed as

1 $$R=-\,\frac{\partial }{\partial x}({\mu }_{p}Ep-{D}_{p}\frac{\partial p}{\partial x})=-\,{\mu }_{p}\frac{\partial }{\partial x}({E}_{0}{p}_{0}+{\rm{\Delta }}E{p}_{0}+{E}_{0}{\rm{\Delta }}p+{\rm{\Delta }}E{\rm{\Delta }}p)+{D}_{p}\frac{{\partial }^{2}{\rm{\Delta }}p}{\partial {x}^{2}}$$

On the other hand, Auger recombination is neglected in the analytical model due to weak excitation, and this assumption can be justified in comparison with simulation results. The Shockley-Reed-Hall theory of recombination under low-level excitation (*n*_0_ ≫ Δ*n*, Δ*p*) is adopted so that *R* = Δ*p*/*τ*, where *τ*, the minority carrier lifetime, is a constant which depends on the trap level and capture cross section^28^. The spatial derivative of the term *E*_0_*p*_0_ in Eq. (1) is zero, and the spatial derivatives of the terms Δ*Ep*_0_ and Δ*E*Δ*p* are assumed to be negligible due to appropriate electrical bias and weak optical excitation. After the solutions for Δ*E* and Δ*p* are found, this assumption can be justified. Then the continuity equation for minority carriers becomes

2 $$\frac{{\rm{\Delta }}p}{\tau }=-\,{\mu }_{p}\frac{\partial }{\partial x}({E}_{0}{p}_{0}+{\rm{\Delta }}E{p}_{0}+{E}_{0}{\rm{\Delta }}p+{\rm{\Delta }}E{\rm{\Delta }}p)+{D}_{p}\frac{{\partial }^{2}{\rm{\Delta }}p}{\partial {x}^{2}}\approx -\,{\mu }_{p}{E}_{0}\frac{\partial {\rm{\Delta }}p}{\partial x}+{D}_{p}\frac{{\partial }^{2}{\rm{\Delta }}p}{\partial {x}^{2}}$$

The solution to Eq. (2) is then given by $\,\bar{p}{e}^{-(x-{x}_{pump})/{L}_{p,\pm }}$, simple exponential decay functions with the coefficient of the minority carrier concentration $\bar{p}$, the decay length *L*_*p*,+_ for the cathode region, and *L*_*p*,−_ for the anode region with respect to the excitation position^29^,

3 $${L}_{p,\pm }=\frac{{\mu }_{p}{E}_{0}\pm \sqrt{{\mu }_{p}^{2}{E}_{0}^{2}+4({D}_{p}/{\tau }_{p})}}{2/{\tau }_{p}}=\frac{1}{2}[{L}_{drift,p}\pm \sqrt{{L}_{drift,p}^{2}+4{L}_{diff,p}^{2}}]$$

where the hole drift length *L*_*drift*,*p*_ = *μ*_*p*_*E*_0_*τ* and the hole diffusion length ${L}_{diff,p}=\sqrt{{D}_{p}\tau }$. These two solutions represent the carrier diffusion along and against the carrier drift forced by the electric field^30^. On the other hand, the continuity equation for the majority carriers is more complicated than that for the minority carriers due to the additional non-negligible term Δ*En*_0_. The spatial derivative of the term Δ*E*Δ*n* is negligible in comparison with the derivative of the other two terms Δ*En*_0_ and *E*_0_Δ*n*. The continuity equation for the majority carriers may be written as

$$\begin{array}{rcl}\frac{{\rm{\Delta }}p}{\tau } & = & {\mu }_{n}\frac{\partial }{\partial x}({E}_{0}{n}_{0}+{\rm{\Delta }}E{n}_{0}+{E}_{0}{\rm{\Delta }}n+{\rm{\Delta }}E{\rm{\Delta }}n)+{D}_{n}\frac{{\partial }^{2}{\rm{\Delta }}n}{\partial {x}^{2}}\\  & \approx  & {\mu }_{n}\frac{\partial }{\partial x}({\rm{\Delta }}E{n}_{0}+{E}_{0}{\rm{\Delta }}n)+{D}_{n}\frac{{\partial }^{2}{\rm{\Delta }}n}{\partial {x}^{2}}\end{array}$$

Based on the concept of charge screening, the evolution of the off-equilibrium concentrations is almost governed by the minority carriers^30,31^, and the majority carrier concentration can be calculated. Details of the derivation is presented in the Supplementary Information. The calculated hole and electron distributions under the bias of 1 V, which corresponds to an applied electric field of 2000 V/cm, are shown in Fig. 1b,c, respectively. The majority carrier distribution also resembles simple exponential decay functions. For the case in n-type InAs, the hole concentration in the cathode region has a larger decay length than that in the anode region. For numerical simulation, both Shockley-Read-Hall recombination model and Auger recombination are included. The parameters of InAs nanowire devices used in calculation are *N*_*D*_ = 10^17^ cm^−3^ ^32^, *τ* = 660 ps^33^, *μ*_*n*_ = 4000 cm^2^/V s^34^, *μ*_*p*_ = 60 cm^2^/V s^35^, *L*_*ch*_ = 5 μm, Auger coefficient of 2.2 × 10^−27^ cm^6^/s^36^, and diameter of 200 nm unless otherwise specified. The pumping density *P* is 40 W/cm^2^ and the spot size *w* is as small as 100 nm for comparison with the results of the analytical model unless otherwise specified. The lifetime *τ* is determined experimentally, and thus the influences of trap energy and capture cross section are included. For the range of applied electric field used here, the carrier drift velocity in InAs nanowires shows a linear dependence on the electric field^37^. Therefore, the electric-field dependence of mobility is neglected for InAs nanowire devices in this study. Note that the electron and hole mobilities adopted here are the experimental values from electrical measurements. Thus the effect of impurity scattering is also included. Considering the large diversity of carrier mobility in nanowires due to several factors such as their geometrical dimensions^18,34,38,39^, quality of surface^40–42^, and material imperfections^43^, the simulation results with different hole mobilities for the verification of model effectiveness will be also presented. However, due to the lack of appropriate carrier-carrier scattering model in InAs, its effect on mobility is not considered in the simulation. Later the simulation will be also carried out for silicon, whose mobility model is more comprehensive. For example, lattice scattering, impurity scattering, and carrier-carrier scattering are all included for the mobility model in Si^44^. An electric-field-dependent mobility model for Si is also adopted^45^. The carrier distribution calculated by the analytical model fits well with the simulation results except for regions near the electrodes, where no excess carriers exist.

With the boundary condition that voltage difference between electrodes is fixed at the bias level, and after lengthy calculation detailed in the Supplementary Information, the photocurrent profile as a function of excitation position *x*_*pump*_ can be derived as

4 $${\rm{\Delta }}J({x}_{pump})\approx \frac{{q}^{2}{\mu }_{n}{n}_{0}}{\varepsilon {L}_{ch}}[-\alpha {e}^{\frac{(-{x}_{pump})}{{L}_{anode,n}}}-\beta {e}^{\frac{(-{x}_{pump})}{|{L}_{p,-}|}}-\gamma {e}^{\frac{{x}_{pump}}{{L}_{p,+}}}+\delta ]$$

where parameters *α*, *β*, *γ*, *δ*, *L*_*anode*,*n*_, *L*_*p*,−_, and *L*_*p*,+_ are all independent of *x*_*pump*_ and the expressions of parameters *α*, *β*, *γ*, *δ*, *L*_*anode*,*n*_, are given in the Supplementary Information. All terms in the expression of Δ*J* have been lumped together according to their different decay lengths. The normalized scanning photocurrent spatial profile calculated using Eq. (4) is shown in Fig. 1d. The result from numerical simulation is also shown in this figure, and it is consistent with the proposed analytical model. Therefore, the assumption of negligible Auger recombination for the analytical model is justified. The same conclusion can be drawn from the simulation for Si nanowires (see Supplementary Information Fig. S1). For the minority carrier distribution, it is plausible to assume that photo-carrier-induced electric field Δ*E* is negligible like most text books did^29–31^. On the other hand, the contribution of Δ*E* is crucial when it comes to the photocurrent profile. The photocurrent density Δ*J* can be decomposed into the drift current and the diffusion current contributed by both carriers and further approximated under weak excitation as in the following

$$\begin{array}{rcl}\frac{{\rm{\Delta }}J}{q} & = & {\mu }_{n}({E}_{0}{\rm{\Delta }}n+{\rm{\Delta }}E{n}_{0}+\Delta E{\rm{\Delta }}n)+{D}_{n}\frac{d\Delta n}{dx}+{\mu }_{p}({E}_{0}{\rm{\Delta }}p+{\rm{\Delta }}E{p}_{0}+{\rm{\Delta }}E{\rm{\Delta }}p)-{D}_{p}\frac{d{\rm{\Delta }}p}{dx}\\  & \approx  & {\mu }_{n}({E}_{0}{\rm{\Delta }}n+{\rm{\Delta }}E{n}_{0})+{D}_{n}\frac{d{\rm{\Delta }}n}{dx}+{\mu }_{p}{E}_{0}{\rm{\Delta }}p-{D}_{p}\frac{d{\rm{\Delta }}p}{dx}\end{array}$$

The large value of the majority carrier concentration *n*_0_ leads to a non-negligible contribution of the term Δ*En*_0_ to photocurrent density even when Δ*E* is much smaller than *E*_0_. It should be noted that from exponential functions Δ*n* and Δ*p* without the term Δ*En*_0_, it will not be possible to obtain a nontrivial Δ*J* which is a constant in space according to the continuity equations under the steady state condition. Numerical simulation and the derived analytic formula both generate almost identical photocurrent profiles in Fig. 1d which are dramatically different from the simple exponential decay function, *i*. *e*., the scanning photocurrent profile and the carrier distribution do not share the same functional form under this weak excitation condition. In order to extract the diffusion length *L*_*diff*,*p*_ and the drift length *L*_*diff*,*p*_ from the photocurrent profile through Eq. (3) unambiguously, we focus on the term with the decay length *L*_*p*,+_ in Eq. (4) which is the dominant term in the cathode region except the constant term *δ*. The hole decay length *L*_*p*,+_ in n-type materials can be simply extracted by fitting the measured photocurrent profiles on the cathode side with a fitting function

5 $$a-b{e}^{x/c}$$

where the symbols *a*, *b*, and *c* are the fitting parameters. The obtained parameter *c* represents the fitted decay length, a positive value.

**Figure 2:**
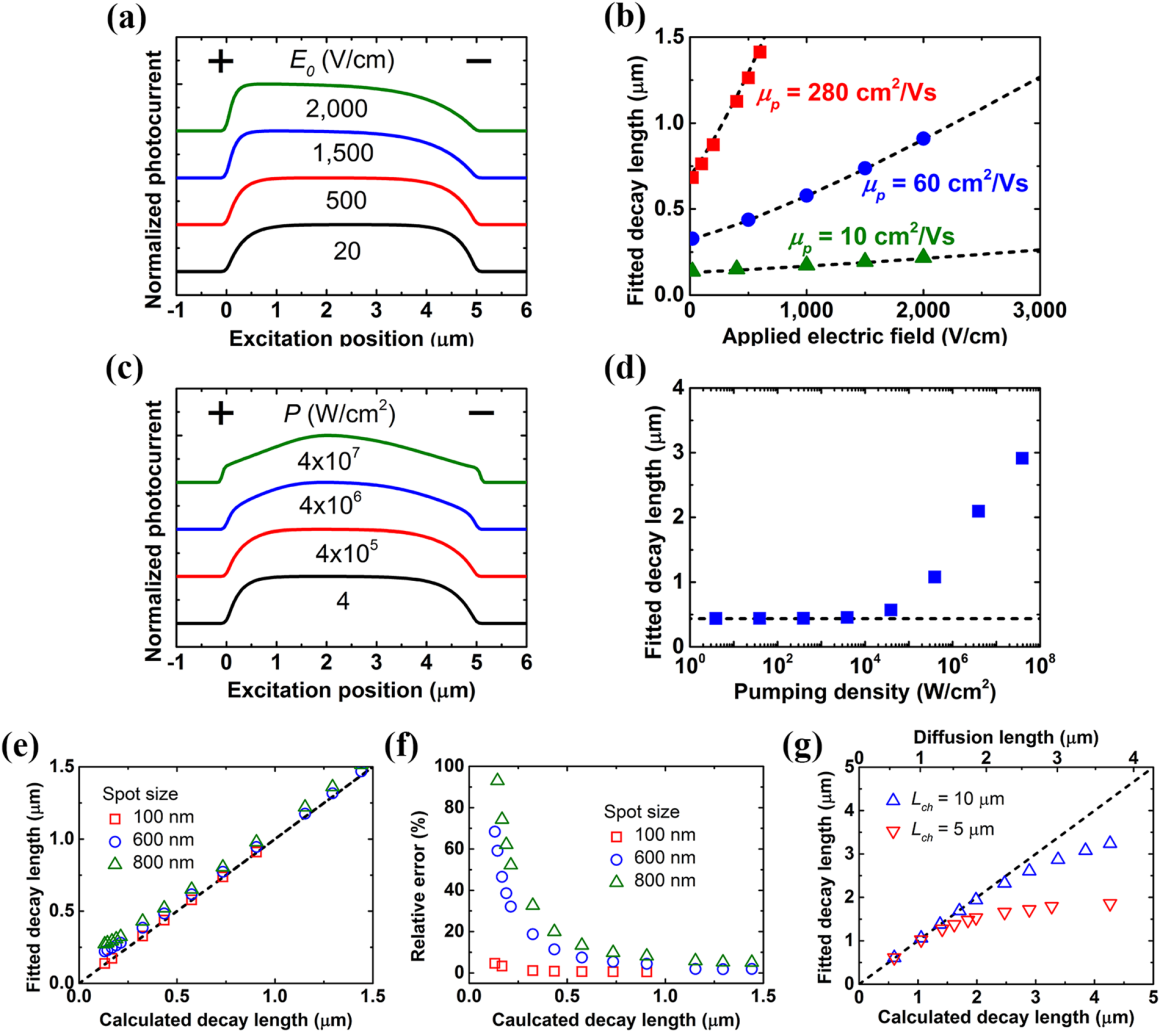
Effectiveness of the analytical model for n-type InAs nanowires. (**a**) Scanning photocurrent profiles with varied applied electric field. (**b**) Fitted decay length as a function of applied electric field, (**c**) Scanning photocurrent profiles with varied pumping density. (**d**) Fitted decay length as a function of pumping density. (**e**) The relations between the fitted decay length and the calculated decay length with varied excitation spot size. (**f**) The relative error of the fitted decay length as a function of calculated decay length with varied excitation spot size. (**g**) Fitted decay length as a function of the calculated decay length with varied *L*_*ch*_. The applied electric field is 20 V/cm, and the calculated decay length is varied by changing the hole mobility. The corresponding diffusion length is also shown as top x axis. The applied electric field for simulation in (**c**,**d**) is set to be 500 V/cm. The dashed lines in (**b**,**d**) indicate the calculated decay lengths using Eq. (3). The *x* = *y* line is shown in (**e**,**g**) as the dashed line for clarity. The photocurrent profiles in (**a**,**c**) are offset vertically for clarity.

After the derivation of the analytic formula for scanning photocurrent profiles, the effectiveness of the proposed analytical model for scanning photocurrent measurements will be verified with the help of numerical simulation. Then the simulated scanning photocurrent profiles are fitted using the analytic formula. Figure 2 shows the fitted decay length based on the fitting function of Eq. (5) for different nanowire devices under various operation conditions. Figure 2a,b shows the scanning photocurrent profiles and the corresponding fitted decay length with varied applied electric field across the nanowires, respectively. The dashed line in Fig. 2b indicates the calculated decay length *L*_*cal*_ = *L*_*p*,+_ using Eq. (3). The decay length is expected to have positive correlation with the applied bias. Therefore, we can investigate the electric-field dependent transport properties in widely used ohmic-contact nanowire devices. The fitted decay length is well consistent with the calculated decay length in a wide range of electric field from 20 to 2000 V/cm. The results with different hole mobilities of 10 and 280 cm^2^/Vs are also presented in Fig. 2b^46^. The fitted decay length is in agreement with the calculated decay length for all cases in that figure. The photocurrent profiles and the fitted decay length with varied pumping density are shown in Fig. 2c,d. The applied electric field for simulation is set to be 500 V/cm, and the calculated decay length *L*_*cal*_ = *L*_*p*,+_ using Eq. (3) is shown as the dashed line in Fig. 2d. It is found that the photocurrent profile and the corresponding fitted decay length remain almost unchanged for pumping density from 4 up to 4 × 10^4^ W/cm^2^, which corresponds to a peak accumulated photo-carrier concentration of slightly smaller than the ionized impurity concentration *N*_*D*_. For pumping density larger than 4 × 10^5^ W/cm^2^, where peak photo-carrier concentrations are higher than *N*_*D*_ of 10^17^ cm^−3^, the fitted decay length deviates largely from the calculated one. The fitting fails for stronger pumping due to the invalid assumption *n*_0_ ≫ Δ*n*, Δ*p* under this condition. Similar results have been observed for Si nanowires (see Supplementary Information Fig. S2). Note that the photocurrent profile for Si nanowires experiences a fundamental change as pumping density increases. The photocurrent profile resembles a simple exponential decay function rather than Eq. (5) for large pumping density as high as 7 × 10^5^ W/cm^2^. In that case, the peak accumulated photo-carrier concentration will be much higher than the designated doping concentration of 10^14^ cm^−3^ in Si nanowires. Later, a separate section will be dedicated to the discussion of the strong excitation condition. Figure 2e shows the relation between the fitted decay length and the calculated decay length. The operation points are sampled from results in Fig. 2b. The influence of excitation spot size on this relation is also demonstrated. The pumping density for spot sizes of 600 nm and 800 nm are set to maintain the same total number of absorbed photons as that for spot size of 100 nm. With the guide of the *x* = *y* line shown as the dashed line in Fig. 2e, we found that the fitted decay length gradually differs from the calculated value with larger excitation spot size. This implies that the higher accuracy of the carrier decay length extraction can be achieved by reducing the excitation spot size through, for example, adopting pump lasers with shorter emission wavelength. The relative error of the fitted decay length is shown in Fig. 2f. The relative error is defined as |*L*_*fit*_ − *L*_*cal*_|/*L*_*cal*_ × 100%, where *L*_*fit*_ is the fitted decay length and *L*_*cal*_ is the calculated decay length. The calculated decay length is the theoretical value in our model. It is worth noting that the relative error increases with larger excitation spot size or decreasing calculated decay length. Besides, the fitted decay length and the calculated decay length are in one-to-one correspondence for a given spot size as shown in Fig. 2e. Thus, with a known excitation spot size, a procedure based on the concept of deconvolution of the measured photocurrent profiles can be performed to obtain the corresponding corrected decay length from the fitted decay length of the experimental photocurrent profile. The corrected decay length should ideally approach the theoretical calculated decay length. This procedure provides an effective correction similar to the powerful technique for correcting the measured linewidths in Raman spectroscopy where the observed spectroscopic band shape is the convolution of the physical and the instrumental contributions^47–49^. To further justify the effectiveness of the analytical model with varied carrier decay length, we plot the fitted decay length as a function of the calculated decay length shown in Fig. 2g. The corresponding diffusion length is also shown as top x axis. The applied electric field is set to be 20 V/cm to keep the calculated decay length close to the diffusion length. For a device with *L*_*ch*_ of 5 μm, a diffusion length up to 1.5 μm can be extracted with error less than 15%. A rule of thumb is that *L*_*ch*_ should be at least about 3 to 4 times longer than the carrier diffusion length for accurate carrier decay length extraction. This is in fact the common practice in SPCM and EBIC literature^4,6,7^.

**Figure 3:**
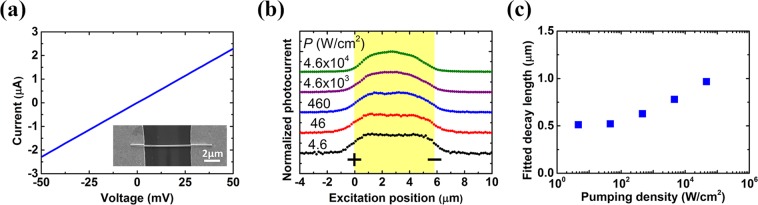
Pumping-density-dependent SPCM measurement in an InAs nanowire. (**a**) I–V characteristics. Inset: SEM image for the measured nanowire device. (**b**) Scanning photocurrent measurement with an applied electric field of 53.1 V/cm under different pumping densities. (**c**) Fitted decay length as a function of pumping density. The nanowire length between electrodes is 5.8 μm shown with yellow background and the excitation spot size is around 800 nm. The photocurrent profiles are offset vertically for clarity.

Now we will apply the analytical model to analyze the experimental SPCM data. Figure 3a shows the current-voltage curves of an InAs nanowire with 5.8 μm in length between electrodes and around 200 nm in diameter (see the Methods for details of the device fabrication). A scanning electron microscopy (SEM) image of the device is shown in the inset. No noticable tapering in the diameter of the nanowire is observed. The resistance of the device is 22 kΩ. The linear relation between the current and the applied voltage shows a typical ohmic contact feature. The line scans of photocurrent along the nanowire are shown in Fig. 3b. Detailed information about SPCM measurement can be found in the Methods. Figure 3c shows the fitted decay length as a function of pumping density. It is noted that the photocurrent profile and the corresponding fitted decay length remain almost unchanged for pumping density below 46 W/cm^2^. Under such weak excitation conditions, the photocurrent profile resembles the fitting function of Eq. (5) instead of a simple exponential decay function. Therefore, the adequacy of the derived analytic formula was confirmed by our experimental SPCM results. For the photocurrent profile with pumping density of 4.6 W/cm^2^, the carrier decay length is extracted to be 520 nm. The value of extracted carrier decay length is over-estimated due to the finite spot size, and the actual carrier decay length is expected to be smaller. Using a cubic polynomial to describe the relation between the fitted decay length and the calculated decay length in Fig. 2e for excitation spot size of about 800 nm in SPCM measurement, we found that the fitted decay length of 520 nm corresponds to a corrected hole decay length of 430 nm.

**Figure 4:**
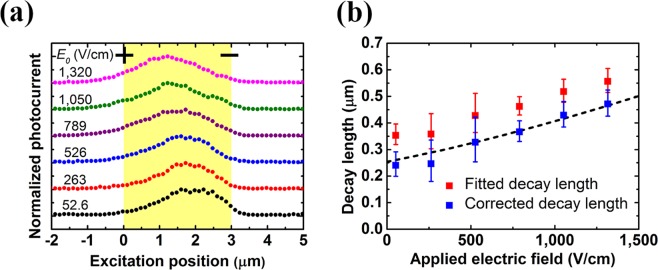
Electric-field-dependent SPCM measurement. (**a**) Scanning photocurrent profiles with varied applied electric field under pumping density of 46 W/cm^2^. (**b**) The fitted and corrected hole decay lengths as a function of the applied electric field. The dashed lines show the fitting curve using Eq. (3). The nanowire length between electrodes is 3 μm shown with yellow background and the excitation spot size is around 800 nm. The photocurrent profiles are offset vertically for clarity.

In order to study the electric-field dependence of the photocurrent profiles, a 3 μm-long device was chosen for investigation. Scanning photocurrent profiles with varied applied electric field under pumping density of 46 W/cm^2^ are shown in Fig. 4a. It is found that the peak of the photocurrent profiles shifts toward the anode as the applied electric field increases. This tendency can be observed in the simulation results in Fig. 2a. In fact, the analytic formula Eq. (4) can also predict this shift. The fitted and corrected hole decay lengths as a function of the applied electric field are depicted in Fig. 4b. The fitted hole decay length can be found with the proposed curve fitting method using Eq. (5). The corresponding corrected decay length is obtained from the fitted decay length using the results in Fig. 2e. By fitting Eq. (3) with the corrected decay length as the decay length *L*_*p*,+_, together with the application of Einstein relation, the mobility-lifetime product is extracted to be 2.5 × 10^−8^ cm^2^/V, and the corresponding diffusion length is 250 nm. The obtained diffusion length is about 50% higher than the hole diffusion length measured in GaAs nanowire p-n junctions^7^. While InAs has a bulk hole mobility somewhat larger than that of GaAs, the higher doping density in the GaAs nanowire p-n junctions may also contribute to the difference between these two experimental results.

**Figure 5:**
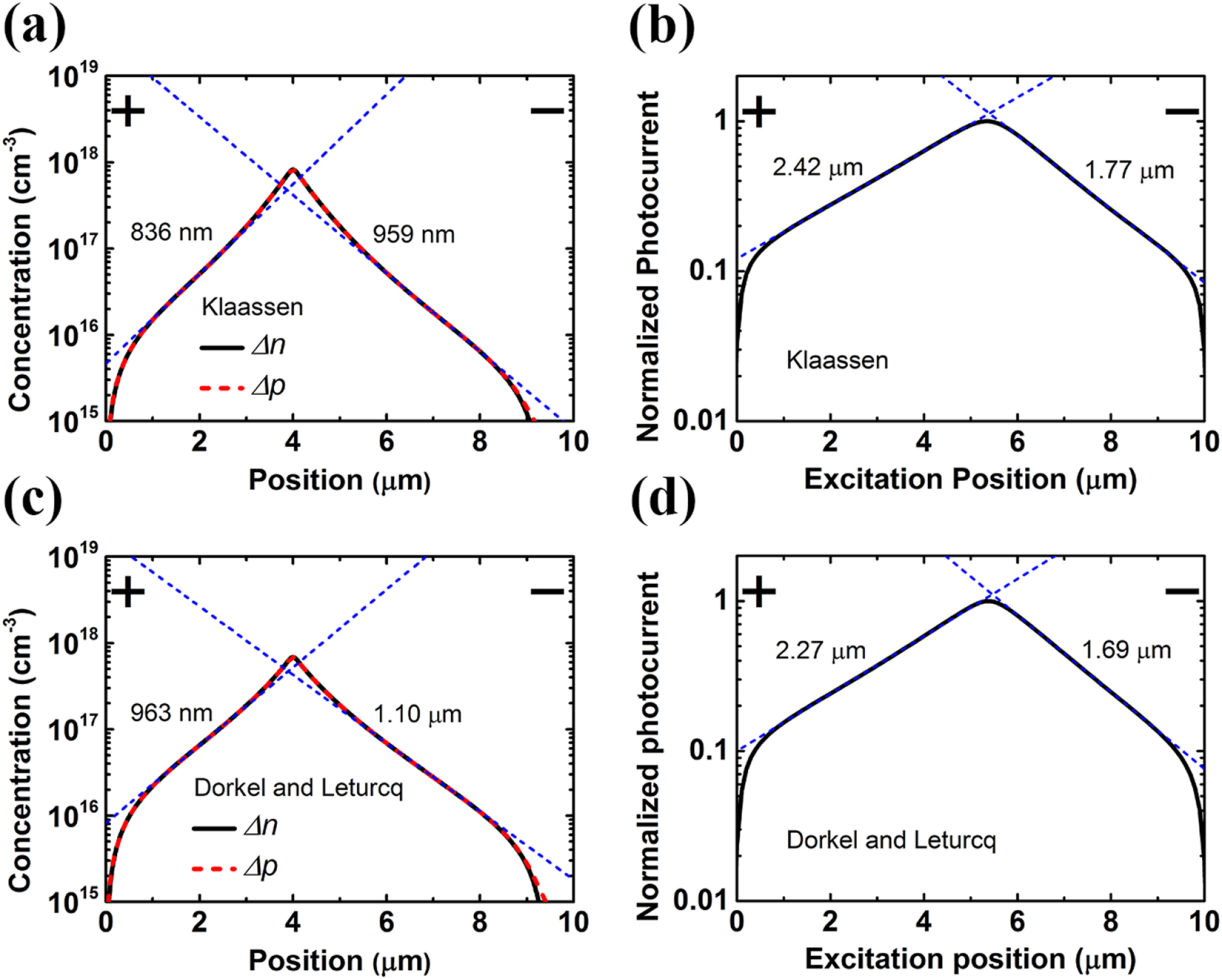
(**a**,**c**) Simulated spatial distribution of photo-carrier-induced electron concentration and hole concentration for n-type Si nanowires under strong excitation. The excitation is centered at 4 μm. Decay lengths are 959/836 nm for (**a**) and 1100/963 nm for (**c**) in the cathode/anode regions, respectively. (**b**,**d**) Scanning photocurrent profiles. The corresponding photocurrent decay lengths are 1.77/2.42 μm for (**b**) and 1.69/2.27 μm for (**d**) on the cathode/anode sides, respectively. The mobility model proposed by Klaassen is used in (**a**,**b**), and Dorkel-Leturcq model is adopted in (**c**,**d**). The applied electric field of the device is 500 V/cm and the pumping density is 7 × 10^5^ W/cm^2^. The fitting curves using simple exponential decay functions are shown as dashed blue lines and corresponding fitted lengths on both sides are also shown in the figures.

Up to now, the proposed analytical model has been validated by numerical simulation, and we have also confirmed the adequacy of the analytic formula experimentally. A new fitting method using Eq. (5) can be applied to extract the carrier decay length from the measured scanning photocurrent profile under weak excitation in ohmic-contact nanowire devices. However, also from nanowire devices with ohmic contact on both sides, photocurrent profiles which resemble simple exponential decay functions were reported experimentally in the SPCM literature^1,3,9^. In this section, we discuss the simulated scanning photocurrent profiles for a nanowire under strong excitation (Δ*n*, Δ*p* ≫ *n*_0_), where the analytical model cannot apply. Under this excitation condition, the accumulated photo-carrier concentrations are large, and the effect of carrier-carrier scattering may not be negligible. Thus the numerical simulation is performed for ohmic-contact nanowire devices of silicon, whose mobility model is more comprehensive than other semiconductor materials. Figure 5 shows the simulated results for a silicon nanowire device with pumping density of 7 × 10^5^ W/cm^2^. Lattice scattering, impurity scattering, and carrier-carrier scattering are all included for the mobility model. Two common models adopted in the simulation were proposed by Klaassen^44^ and by Dorkel and Leturcq^50^. An electric-field-dependent mobility model is also adopted^45^. Both Shockley-Read-Hall recombination model and Auger recombination are included in the numerical simulation. Parameters used for the silicon nanowire device are *L*_*ch*_ = 10 μm, *N*_*D*_ = 10^14^ cm^−3^, and *τ* = 420 ps^51^. The applied electric field is 500 V/cm, the nanowire diameter is 100 nm, and the spot size is set to be 100 nm. Auger coefficients are 2.8 × 10^−31^ and 9.9 × 10^−32^ cm^6^/s for electrons and holes, respectively^52^. As shown in Fig. 5a,c, the peak accumulated photo-carrier concentrations with the pump centered at 4 μm is found to be about 10^18^ cm^−3^, which is much higher than *N*_*D*_. The fitted decay lengths of the exponentially decayed electron/hole concentration (959 and 1100 nm in the cathode region for Klaassen and Dorkel-Leturcq models, respectively) are extracted from the simulated carrier distributions. The SPCM profiles with Klaassen and Dorkel-Leturcq models are shown in Fig. 5b,d. Both profiles resemble simple exponential decay functions similar to the experimental results observed in the SPCM literature^1,3,9^. However, the fitted photocurrent decay lengths using simple exponential decay functions (1.77 and 1.69 μm on the cathode side for Klaassen and Dorkel-Leturcq models, respectively) are found to be remarkably different from the fitted electron/hole decay length. Obviously, the scanning photocurrent profiles do not share the same decay lengths with electron/hole distributions. Thus, it is concluded that carrier decay length extraction from such photocurrent decay profiles under strong excitation can suffer large inaccuracy in ohmic-contact nanowire devices.

## Conclusion

An analytic formula for scanning photocurrent profiles in ohmic-contact nanowire devices under uniform applied electric field and weak excitation is derived. The influence of photo-carrier-induced electric field Δ*E* is included when solving coupled Poisson’s and continuity equations for photocurrent. It is found that the scanning photocurrent profile and the carrier spatial distribution do not share the same functional form under weak excitation. Instead, a surprising new analytic relation between the scanning photocurrent profile and the minority carrier decay length was established. The analytic formula was validated by numerical simulations in InAs and Si nanowires. The effectiveness of the formula in nanowires with different mobility values under different pumping and bias conditions was thoroughly discussed. The experimental photocurrent profiles also confirmed the adequacy of the derived analytic formula. Electric-field dependence of the carrier decay length was directly observed. Then the mobility-lifetime product and the carrier diffusion length were obtained. The photocurrent profiles of ohmic-contact nanowire devices in strong excitation regime were also investigated using numerical simulation, and they resembled simple exponential decay functions which are similar to those reported experimentally in the SPCM literature. However, the fitted photocurrent decay length was found to be remarkably different from the fitted carrier decay length. Carrier decay length extraction from such photocurrent decay profiles under strong excitation can suffer large inaccuracy in ohmic-contact nanowire devices. On the other hand, the derived analytic formula in this work provides a new simple fitting method to correctly determine the minority carrier decay length. With this formula, we will be able to quantitatively evaluate the performance of nanowire-based devices with a single scanning photocurrent or electron-beam-induced current measurement setup.

## Methods

### Device fabrication

The InAs nanowires in this study were grown on a Si (111) substrate by the molecular beam epitaxy (MBE) technique. Before the growth, a 100-nm-thick SiO_2_ layer was deposited on the substrate by plasma-enhanced chemical vapor deposition (PECVD). Nanowires were deposited on the oxide-coated substrate directly. The V/III ratio and growth temperature were 400 and 420 °C, respectively. The InAs nanowires are about 6–10 μm in length and 200–230 nm in diameter. Si substrates with 200 nm thick SiO_2_ grown by PECVD were prepared for mechanical nanowire transfer. Prior to metal evaporation, the native oxide of the InAs nanowires was removed to ensure a good ohmic contact between metal and nanowires. Electron-gun evaporation was used to deposit titanium/gold film and then followed by a lift-off process. Finally, rapid thermal annealing was performed to improve the metal-semiconductor contact quality.

### Scanning photocurrent microscopy

A 532 nm continuous-wave laser was modulated with a chopper and focused onto an InAs nanowire by a 100X objective lens for local excitation of the device with excitation spot size of about 800 nm. The excitation position was precisely controlled using piezo-electric manipulators. A source meter unit was used to apply bias across the nanowire, and the photo-carrier-induced current was measured with a lock-in amplifier.

## Supplementary information

Supplementary Information

## Data Availability

The data reported in this paper are available upon request.
