# Supplementary material for: A New Analytic Formula for Minority Carrier Decay Length Extraction from Scanning Photocurrent Profiles in Ohmic-Contact Nanowire Devices
Source: Sci Rep. 2019 Jul 1;9:9426. doi: 10.1038/s41598-019-46020-2 (PMC6603194; doi:10.1038/s41598-019-46020-2)
Supplement: Supplementary file 1 — Supplementary Information [file 41598_2019_46020_MOESM1_ESM.pdf]

Supplementary Information

A New Analytic Formula for Minority Carrier Decay Length Extraction from Scanning Photocurrent Profiles in Ohmic-Contact Nanowire Devices

Cheng-Hao Chu,[1] Ming-Hua Mao,[1,2,3,*] Che-Wei Yang,[1] and Hao-Hsiung Lin[1,2,3]

[1]Graduate Institute of Electronics Engineering, National Taiwan University, No. 1, Roosevelt Rd. Sec. 4, Taipei 10617, Taiwan

[2]Department of Electrical Engineering, National Taiwan University, No. 1, Roosevelt Rd. Sec. 4, Taipei 10617, Taiwan

[3]Graduate Institute of Photonics and Optoelectronics, National Taiwan University, No. 1, Roosevelt Rd. Sec. 4, Taipei 10617, Taiwan

*Correspondence and requests for materials should be addressed to M.H.M. (email: mhmao@ntu.edu.tw)

Derivation of the analytic formula for scanning photocurrent profiles. The proposed analytical model for scanning photocurrent microscopy (SPCM) under electrical bias and weak optical excitation is based on Poisson's equation and continuity equations for electrons and holes.[1,2] Consider one-dimensional transport in a two-terminal Ohmic-contact nanowire device with the anode at $x = 0$ and the cathode at $x = L_{ch}$, where L_{ch} is the nanowire length between electrodes. We may write

$$\frac{q}{\varepsilon}(p - n + N_D - N_A) = \frac{\partial E}{\partial x}$$

$$\frac{\partial n}{\partial t} = \frac{1}{q}\frac{\partial J_n}{\partial x} + G - R = 0,\ J_n = q\mu_n E n + qD_n\frac{\partial n}{\partial x}$$

$$\frac{\partial p}{\partial t} = -\frac{1}{q}\frac{\partial J_p}{\partial x} + G - R = 0,\ J_p = q\mu_p E p - qD_p\frac{\partial p}{\partial x}$$

where n and p are the electron and hole concentrations. N_D and N_A are the ionized donor and acceptor concentrations, respectively. Uniform dopants are assumed in this study. E is the electric field, q is the fundamental charge, and R is the net recombination rate. J_n and J_p are the electron and hole current densities, respectively. $\mu_{n,p}$ and $D_{n,p}$ are the electron/hole mobilities and diffusion coefficients of the nanowire, respectively. For carrier transport in the steady state, the time derivatives of the electron and hole concentrations are both zero. The generation rate G by optical excitation in this model is assumed to be a delta function centered at x_{pump}.

When the nanowire is optically pumped, the electron concentration, the hole concentration, and the electric field differ from their values without excitation. These variables can be expressed as their values without excitation plus the optical-excitation-induced changes, i.e. $n = n_0 + \Delta n$, $p = p_0 + \Delta p$, and $E = E_0 + \Delta E$, where n_0, p_0, and E_0 are the values without excitation and Δn, Δp, and ΔE are their optical-excitation-induced changes. An n-type semiconductor nanowire is taken here as an example. Thus, $N_D \gg N_A$ is assumed in Poisson's equation. The continuity equation for minority

carriers is given by

$$R = -\frac{\partial}{\partial x}\left(\mu_p E p - D_p \frac{\partial p}{\partial x}\right) = -\mu_p \frac{\partial}{\partial x}(E_0 p_0 + \Delta E p_0 + E_0 \Delta p + \Delta E \Delta p) + D_p \frac{\partial^2 \Delta p}{\partial x^2} \tag{S1}$$

On the other hand, Auger recombination is neglected in the analytical model due to weak excitation, and the Shockley-Reed-Hall theory of recombination under low-level excitation ($n_0 \gg \Delta n, \Delta p$) is adopted so that $R = \Delta p/\tau$, where τ, the minority carrier lifetime, is constant.[3] The spatial derivative of the term $E_0 p_0$ in equation (S1) is zero, and the spatial derivatives of the terms $\Delta E p_0$ and $\Delta E \Delta p$ are assumed to be negligible due to appropriate electrical bias and weak optical excitation. After the solutions for ΔE and Δp are found, this assumption can be justified. Then the continuity equation for minority carriers becomes

$$\frac{\Delta p}{\tau} = -\mu_p \frac{\partial}{\partial x}(E_0 p_0 + \Delta E p_0 + E_0 \Delta p + \Delta E \Delta p) + D_p \frac{\partial^2 \Delta p}{\partial x^2} \approx -\mu_p E_0 \frac{\partial \Delta p}{\partial x} + D_p \frac{\partial^2 \Delta p}{\partial x^2} \tag{S2}$$

The solution to equation (S2) is given by $\Delta p = \bar{p} e^{-(x - x_{pump})/L_{p,\pm}}$ with the coefficient of the minority carrier concentration $\bar{p}$ and the decay length[4]

$$L_{p,\pm} = \frac{\mu_p E_0 \pm \sqrt{\mu_p^2 E_0^2 + 4\left(\frac{D_p}{\tau}\right)}}{\frac{2}{\tau}} = \frac{1}{2}\left[L_{drift,p} \pm \sqrt{L_{drift,p}^2 + 4L_{diff,p}^2}\right]$$

where the hole drift length $L_{drift,p} = \mu_p E_0 \tau$ and the hole diffusion length $L_{diff,p} = \sqrt{D_p \tau}$. These two solutions represent the carrier diffusion along and against the carrier drift forced by the electric field.[5] The positive and the negative signs are taken for the minority carriers in the cathode and the anode region with respect to the excitation position, respectively. In this derivation, the variables including the photo-induced carrier concentrations, the photo-carrier-induced electric field, and the photocurrent density are expressed as piecewise functions in the anode and the cathode regions divided by the excitation position, and we may write

$$\Delta p = \begin{cases} \Delta p_{anode}, & x < x_{pump} \\ \Delta p_{cathode}, & x > x_{pump} \end{cases}$$

The same way of defining piecewise functions in the anode and the cathode regions will be applied to Δn, ΔE, and ΔJ as well. The minority carrier concentrations in each region are

$$\Delta p_{cathode} = \bar{p} e^{-(x - x_{pump})/L_{cathode,p}}$$

and

$$\Delta p_{anode} = \bar{p} e^{(x - x_{pump})/L_{anode,p}}$$

The carrier decay lengths in the cathode and the anode region are given by

$$L_{cathode,p} = L_{p,+} = \frac{1}{2}\left[L_{drift,p} + \sqrt{L_{drift,p}^2 + 4L_{diff,p}^2}\right]$$

and

$$L_{anode,p} = -L_{p,-} = \frac{1}{2}\left[-L_{drift,p} + \sqrt{L_{drift,p}^2 + 4L_{diff,p}^2}\right]$$

On the other hand, the carrier transport of majority carriers is more complicated than that of minority carriers because of the additional non-negligible term $\Delta E n_0$. The spatial derivative of the term $\Delta E \Delta n$ is negligible in comparison with the derivative of the other two terms $\Delta E n_0$ and $E_0 \Delta n$. The continuity equation for the majority carriers may be written as

$$\frac{\Delta p}{\tau} = \mu_n \frac{\partial}{\partial x}(E_0 n_0 + \Delta E n_0 + E_0 \Delta n + \Delta E \Delta n) + D_n \frac{\partial^2 \Delta n}{\partial x^2}$$

$$\approx \mu_n \frac{\partial}{\partial x}(\Delta E n_0 + E_0 \Delta n) + D_n \frac{\partial^2 \Delta n}{\partial x^2} \tag{S3}$$

Due to the charge screening effect, the evolution of the off-equilibrium concentrations is almost governed by the minority carriers,[5,6] and the screening effect becomes stronger with higher minority carrier concentration. As the result, the decay length for electrons in the cathode region will be the same as that for holes in nanowires with large bias (that is, drift dominant case), since $L_{cathode,p}$ is larger and the minority carrier concentration is higher in the cathode region than in the anode region. We may write

$$\Delta n_{cathode} \approx \bar{n} e^{-(x-x_{pump})/L_{cathode,n}}$$

where $\bar{n}$ is the coefficient of the majority carrier concentration. According to Poisson's equation, the spatial derivative of the photo-carrier-induced electric field may be written as

$$\frac{\partial \Delta E}{\partial x} = \frac{q}{\varepsilon}(\Delta p - \Delta n) \tag{S4}$$

Substituting $\Delta n_{cathode}$ and equation (S4) into equation (S3), we have

$$\frac{\Delta p}{\tau} \approx \frac{q\mu_n n_0}{\varepsilon}(\Delta p - \Delta n) - \frac{\mu_n E_0 \Delta n}{L_{cathode,n}} + \frac{D_n \Delta n}{L_{cathode,n}^2}$$

and

$$L_{cathode,n}^2 \left[\frac{q\mu_n n_0}{\varepsilon}(\Delta p - \Delta n) - \frac{\Delta p}{\tau}\right] - L_{cathode,n}\mu_n E_0 \Delta n + D_n \Delta n = 0$$

The solution to the equation above is then given by

$$L_{cathode,n} = \frac{\mu_n E_0 \Delta n \pm \sqrt{(\mu_n E_0 \Delta n)^2 - 4AD_n \Delta n}}{2A}$$

where $A = q\mu_n n_0(\Delta p - \Delta n)/\varepsilon - \Delta p/\tau$. Due to stronger charge screening effect in the cathode region, we simply assume the decay length $L_{cathode,n} = L_{cathode,p} \equiv L_{cathode}$ for drift dominant case, which yield $\mu_n E_0 \Delta n/A = \mu_p E_0 \tau$. Then the ratio between the coefficients of the majority and minority carrier concentrations may be written as

$$\frac{\bar{n}}{\bar{p}} = \left(\frac{q\mu_n\mu_p n_0 \tau}{\varepsilon} - \mu_p\right) \div \left(\frac{q\mu_n\mu_p n_0 \tau}{\varepsilon} + \mu_n\right)$$

Note that though the ratio $\bar{n}/\bar{p}$ is obtained by assuming $L_{cathode,n} = L_{cathode,p} \equiv L_{cathode}$ in the drift dominant regime, the derived analytic formula and the proposed fitting method for scanning photocurrent profiles are verified by numerical simulation in a wide range of bias condition.

Substituting equations (S2) and (S4) into equation (S3), the continuity equation for the majority carriers in the anode region becomes a second-order nonhomogeneous differential equation. We may write

$$D_n \frac{\partial^2 \Delta n_{anode}}{\partial x^2} + (\mu_n E_0)\frac{\partial \Delta n_{anode}}{\partial x} - \left(\frac{q\mu_n n_0}{\varepsilon}\right)\Delta n_{anode} \approx \left(\frac{-\mu_p E_0}{L_{anode,p}} + \frac{D_p}{L_{anode,p}^2} - \frac{q\mu_n n_0}{\varepsilon}\right)\Delta p_{anode} \tag{S5}$$

where $\Delta p_{anode} = \bar{p} e^{(x-x_{pump})/L_{anode,p}}$. We then solve the electron concentration in the anode region, where the complete solution of Δn_{anode} is given by

$$\Delta n_{anode} \approx \overline{n_1} e^{(x-x_{pump})/L_n} + \overline{n_2} e^{(x-x_{pump})/L_{anode,p}}$$

where $L_{anode,p} = |L_{p,-}|$ and $\bar{n} = \overline{n_1} + \overline{n_2}$. The decay length L_n can be solved with the corresponding homogeneous differential equation of equation (S5), which gives

$$\frac{q\mu_n n_0}{\varepsilon} L_n^2 - \mu_n E_0 L_n - D_n = 0$$

The solution to the above equation may be written as

$$L_{n,\pm} = \frac{\mu_n E_0 \pm \sqrt{(\mu_n E_0)^2 + 4\frac{q}{\varepsilon}\mu_n n_0 D_n}}{\frac{2q}{\varepsilon}\mu_n n_0}$$

Note that only the positive sign for $L_{n,\pm}$ is taken here, and we may write

$$\Delta n_{anode} \approx \overline{n_1} e^{(x-x_{pump})/L_{anode,n}} + \overline{n_2} e^{(x-x_{pump})/L_{anode,p}}$$

where $L_{anode,n} = L_{n,+}$. $\overline{n_2}$ is the coefficient of the particular solution to equation (S5), which may be written as

$$\frac{\overline{n_2}}{\bar{p}} = \frac{\frac{q}{\varepsilon}\left(L_{anode,p}^2 \mu_n n_0\right) + \mu_p E_0 L_{anode,p} - D_p}{\frac{q}{\varepsilon}\left(L_{anode,p}^2 \mu_n n_0\right) - \mu_n E_0 L_{anode,p} - D_n}$$

With the relation $\bar{n} = \overline{n_1} + \overline{n_2}$, the coefficient $\overline{n_1}$ is then given by

$$\frac{\overline{n_1}}{\bar{p}} = \frac{\frac{q}{\varepsilon}(\bar{n}-\bar{p})\left(L_{anode,p}^2 \mu_n n_0\right) - (\mu_p \bar{p} + \mu_n \bar{n}) E_0 L_{anode,p} + D_p \bar{p} - D_n \bar{n}}{\bar{p}\left[\frac{q}{\varepsilon}\left(L_{anode,p}^2 \mu_n n_0\right) - \mu_n E_0 L_{anode,p} - D_n\right]}$$

According to Poisson's equation, the photo-carrier-induced electric field in the anode region is given by

$$\Delta E_{anode}(x) = \frac{q}{\varepsilon}\int_0^x (\Delta p - \Delta n)dx' + \overline{\Delta E}$$

$$\approx \frac{q}{\varepsilon}\left[L_{anode,p}(\bar{p} - \overline{n_2})e^{\frac{x-x_{pump}}{L_{anode,p}}} - L_{anode,p}(\bar{p} - \overline{n_2})e^{\frac{-x_{pump}}{L_{anode,p}}}\right]$$

$$+\frac{q}{\varepsilon}\left[-L_{anode,n}\overline{n_1}e^{\frac{x-x_{pump}}{L_{anode,n}}} + L_{anode,n}\overline{n_1}e^{\frac{-x_{pump}}{L_{anode,n}}}\right] + \overline{\Delta E}$$

where $\overline{\Delta E}$ is the constant of integration for the photo-carrier-induced electric field. The photocurrent density in the anode region may be written as

$$\frac{\Delta J_{anode}}{q} = \mu_n(E_0 \Delta n + \Delta E_{anode} n_0 + \Delta E_{anode} \Delta n) + D_n \frac{d\Delta n}{dx}$$

$$+\mu_p(E_0 \Delta p + \Delta E_{anode} p_0 + \Delta E_{anode} \Delta p) - D_p \frac{d\Delta p}{dx}$$

$$\approx \mu_n(E_0 \Delta n + \Delta E_{anode} n_0) + D_n \frac{d\Delta n}{dx} + \mu_p E_0 \Delta p - D_p \frac{d\Delta p}{dx}$$

$$\approx e^{\frac{x-x_{pump}}{L_{anode,n}}}\left[\mu_n E_0 \overline{n_1} - \frac{q L_{anode,n}\overline{n_1}}{\varepsilon}(\mu_n n_0) + \frac{D_n \overline{n_1}}{L_{anode,n}}\right]$$

$$+e^{\frac{x-x_{pump}}{L_{anode,p}}}\left[\frac{q L_{anode,p}(\bar{p}-\overline{n_2})}{\varepsilon}(\mu_n n_0) + E_0\left(\mu_n \overline{n_2} + \mu_p \bar{p}\right) + \frac{D_n \overline{n_2} - D_p \bar{p}}{L_{anode,p}}\right]$$

$$+\frac{q}{\varepsilon}\left[L_{anode,n}\overline{n_1}e^{\frac{-x_{pump}}{L_{anode,n}}} - L_{anode,p}(\bar{p} - \overline{n_2})e^{\frac{-x_{pump}}{L_{anode,p}}}\right](\mu_n n_0) + \overline{\Delta E}\mu_n n_0$$

Substituting the solved parameters $L_{anode,n}$, $L_{anode,p}$, and $\overline{n_2}$, into the above equation, we find that the coefficients of the both terms $e^{(x-x_{pump})/L_{anode,n}}$and $e^{(x-x_{pump})/L_{anode,p}}$ are zero, which gives

$$\frac{\Delta J_{anode}}{q} \approx \frac{q}{\varepsilon}\left[L_{anode,n}\overline{n_1}e^{\frac{-x_{pump}}{L_{anode,n}}} - L_{anode,p}(\bar{p} - \overline{n_2})e^{\frac{-x_{pump}}{L_{anode,p}}}\right](\mu_n n_0) + \overline{\Delta E}\mu_n n_0$$

The same treatment can also be performed in the cathode region, which yields

$$\Delta E_{cathode}(x) = \frac{q}{\varepsilon}\int_0^{x_{pump}}(\Delta p - \Delta n)dx' + \Delta E_{source} + \frac{q}{\varepsilon}\int_{x_{pump}}^x (\Delta p - \Delta n)dx' + \overline{\Delta E}$$

$$\approx \frac{q}{\varepsilon}\left[1 - e^{\frac{-(x-x_{pump})}{L_{cathode}}}\right]L_{cathode}(\bar{p} - \bar{n}) + \Delta E_{anode}(x_{pump}) + \Delta E_{source}$$

and

$$\frac{\Delta J_{cathode}}{q} \approx e^{\frac{-(x-x_{pump})}{L_{cathode}}}\left\{E_0\left(\mu_n\bar{n}+\mu_p\bar{p}\right)-\frac{qL_{cathode}}{\varepsilon}(\mu_n n_0)(\bar{p}-\bar{n})+\frac{D_p\bar{p}-D_n\bar{n}}{L_{cathode}}\right\}$$

$$+\frac{q}{\varepsilon}\left[L_{cathode}(\bar{p}-\bar{n})+L_{anode,p}(\bar{p}-\overline{n_2})-L_{anode,n}\overline{n_1}\right](\mu_n n_0)$$

$$+\frac{q}{\varepsilon}\left[L_{anode,n}\overline{n_1}e^{\frac{-x_{pump}}{L_{anode,n}}}-L_{anode,p}(\bar{p}-\overline{n_2})e^{\frac{-x_{pump}}{L_{anode,p}}}\right](\mu_n n_0)$$

$$+(\overline{\Delta E}+\Delta E_{source})\mu_n n_0$$

where ΔE_{source} is the photo-carrier-induced electric field at excitation position. The coefficient of the term $e^{-(x-x_{pump})/L_{cathode}}$ is zero according to the continuity equations. Under the steady state condition, the photocurrent density ΔJ must be a single value throughout the whole space. Comparing two results of ΔJ in the anode and the cathode region, we obtain

$$\Delta E_{source}=\frac{q}{\varepsilon}\left[-L_{cathode}(\bar{p}-\bar{n})-L_{anode,p}(\bar{p}-\overline{n_2})+L_{anode,n}\overline{n_1}\right]$$

The global expression of photocurrent density is then given by

$$\Delta J \approx \frac{q^2}{\varepsilon}\left[L_{anode,n}\overline{n_1}e^{\frac{-x_{pump}}{L_{anode,n}}}-L_{anode,p}(\bar{p}-\overline{n_2})e^{\frac{-x_{pump}}{L_{anode,p}}}\right](\mu_n n_0)+q\overline{\Delta E}\mu_n n_0 \qquad \text{(S6)}$$

The last unknown variable for solving the scanning photocurrent profiles is the constant of integration for the photo-carrier-induced electric field $\overline{\Delta E}$. The total photo-carrier-induced voltage drop across the anode and the cathode regions can be written as

$$-\Delta V_{anode}=\int_0^{x_{pump}}\Delta E\,dx$$

$$\approx\frac{q}{\varepsilon}\left[(\bar{p}-\overline{n_2})L_{anode,p}^2-\overline{n_1}L_{anode,n}^2\right]$$

$$+\frac{q}{\varepsilon}e^{\frac{(-x_{pump})}{L_{anode,p}}}(\bar{p}-\overline{n_2})\left(-L_{anode,p}^2-x_{pump}L_{anode,p}\right)$$

$$+\frac{q}{\varepsilon}e^{\frac{(-x_{pump})}{L_{anode,n}}}\overline{n_1}\left(L_{anode,n}^2+x_{pump}L_{anode,n}\right)+\overline{\Delta E}x_{pump}$$

and

$$-\Delta V_{cathode}=\int_{x_{pump}}^{L_{ch}}\Delta E\,dx$$

$$\approx\frac{q}{\varepsilon}\left[(\bar{p}-\overline{n_2})L_{anode,p}-\overline{n_1}L_{anode,n}+(\bar{p}-\bar{n})L_{cathode}\right]\left(L_{ch}-x_{pump}\right)$$

$$+\frac{q}{\varepsilon}\left[-e^{\frac{(-x_{pump})}{L_{anode,p}}}(\bar{p}-\overline{n_2})L_{anode,p}+e^{\frac{(-x_{pump})}{L_{anode,n}}}\overline{n_1}L_{anode,n}\right]\left(L_{ch}-x_{pump}\right)$$

$$+\frac{q}{\varepsilon}(\bar{p}-\bar{n})L_{cathode}^2\left[e^{\frac{-(L_{ch}-x_{pump})}{L_{cathode}}}-1\right]+(\overline{\Delta E}+\Delta E_{source})\left(L_{ch}-x_{pump}\right)$$

Because the potential difference between two electrodes is fixed at the bias level, the photo-carrier-induced potential difference between the electrodes must be zero, and we may write

$$-\Delta V=(-\Delta V_{anode})+(-\Delta V_{cathode})$$

$$\approx\frac{q}{\varepsilon}e^{\frac{(-x_{pump})}{L_{anode,n}}}\left(\overline{n_1}L_{anode,n}^2+\overline{n_1}L_{anode,n}L_{ch}\right)$$

$$+\frac{q}{\varepsilon}e^{\frac{(-x_{pump})}{L_{anode,p}}}\left[-(\bar{p}-\overline{n_2})L_{anode,p}^2-(\bar{p}-\overline{n_2})L_{anode,p}L_{ch}\right]$$

$$+\frac{q}{\varepsilon}e^{\frac{-(L_{ch}-x_{pump})}{L_{cathode}}}(\bar{p}-\bar{n})L_{cathode}^2$$

$$+\frac{qx_{pump}}{\varepsilon}\left[-(\bar{p}-\overline{n_2})L_{anode,p}+\overline{n_1}L_{anode,n}-L_{cathode}(\bar{p}-\bar{n})\right]$$

$$+\frac{q}{\varepsilon}\left[(\bar{p}-\overline{n_2})L_{anode,p}^2+(\bar{p}-\overline{n_2})L_{anode,p}L_{ch}-\overline{n_1}L_{anode,n}^2-\overline{n_1}L_{anode,n}L_{ch}\right]$$

$$+\frac{q}{\varepsilon}\left[L_{cathode}L_{ch}(\bar{p}-\bar{n})-(\bar{p}-\bar{n})L_{cathode}^2\right]+\overline{\Delta E}L_{ch}+\Delta E_{source}\left(L_{ch}-x_{pump}\right)=0$$

The coefficient of the photo-carrier-induced electric field is then given by

$$\overline{\Delta E}\approx\frac{q}{\varepsilon L_{ch}}\left\{-\tilde{\alpha}e^{\frac{(-x_{pump})}{L_{anode,n}}}-\tilde{\beta}e^{\frac{(-x_{pump})}{L_{anode,p}}}-\gamma e^{\frac{x_{pump}}{L_{cathode}}}+\delta\right\}$$

where $\tilde{\alpha}=\overline{n_1}L_{anode,n}\left(L_{anode,n}+L_{ch}\right)$

$\tilde{\beta}=(\overline{n_2}-\bar{p})L_{anode,p}\left(L_{anode,p}+L_{ch}\right)$

$\gamma=L_{cathode}^2e^{\frac{-L_{ch}}{L_{cathode}}}(\bar{p}-\bar{n})$

$\delta=(\overline{n_2}-\bar{p})L_{anode,p}^2+\overline{n_1}L_{anode,n}^2+(\bar{p}-\bar{n})L_{cathode}^2$

Substitute $\bar{E}$ into equation (S6), the photocurrent profile as a function of the excitation position x_{pump} is given by

$$\Delta J\approx\frac{q^2\mu_n n_0}{\varepsilon L_{ch}}\left[-\alpha e^{\frac{(-x_{pump})}{L_{anode,n}}}-\beta e^{\frac{(-x_{pump})}{L_{anode,p}}}-\gamma e^{\frac{x_{pump}}{L_{cathode}}}+\delta\right]$$

where $\alpha=\overline{n_1}L_{anode,n}^2$

$\beta=(\overline{n_2}-\bar{p})L_{anode,p}^2$

Validation of the analytical model for n-type silicon nanowires. Lattice scattering, impurity scattering, and carrier-carrier scattering are all considered for mobility model.[7] An electric-field-dependent mobility model is also adopted.[8] Both Shockley-Read-Hall recombination model and Auger recombination are included in the numerical simulation. Parameters used for the model validation are $L_{ch} = 10\mu m$, $N_D = 10^{14}$ cm^{-3}, and $\tau = 420$ ps.[9] The applied electric field of the device is 500 V/cm, and the nanowire diameter is 100 nm. Auger coefficient of 2.8×10^{-31} and 9.9×10^{-32} cm^6/s for electrons and holes, respectively.[10] The spot size w is as small as 100 nm for comparison with the results of the analytical model.

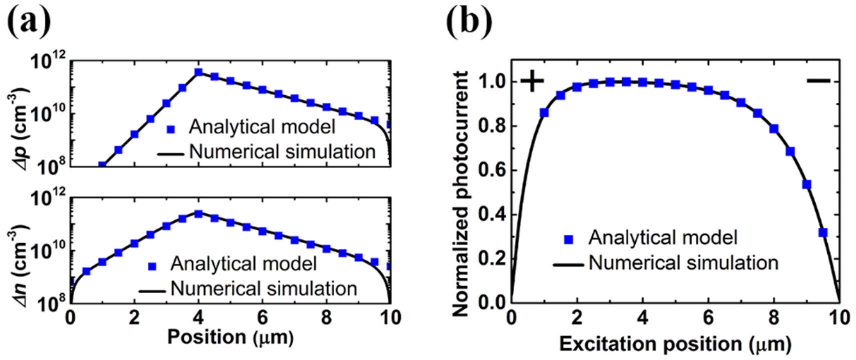

Figure S1. (a) Calculated spatial distribution of photo-induced hole concentration and electron concentration for n-type InAs nanowires with applied electric field of 500 V/cm under excitation at position of 4 μm. (b) Calculated scanning photocurrent profiles. The pumping density P is 0.7 W/cm^2. Results from numerical simulation are also shown for comparison.

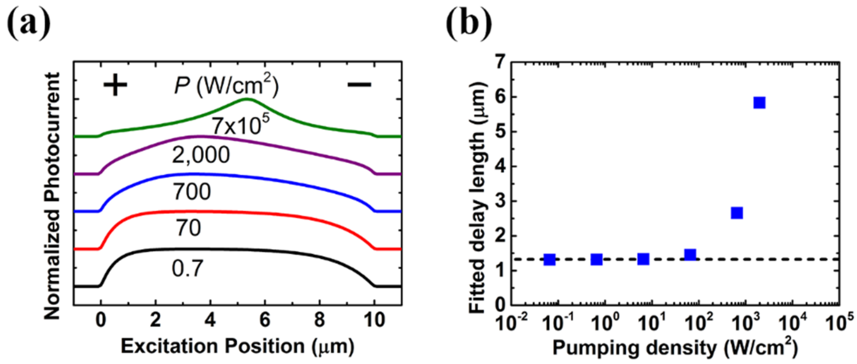

Figure S2. Effectiveness of the proposed analytical model for n-type silicon nanowires. (a) Scanning photocurrent profiles with varied pumping density. The photocurrent profiles are offset vertically for clarity. (b) Fitted decay length as a function of pumping density. The applied electric field is set to be 500 V/cm. The dashed lines in (b) indicate the calculated decay lengths.

References

1. Vasileska, D., Goodnick, S. M. & Klimeck, G. *Computational Electronics*. (CRC Press, 2010).
2. van Roosbroeck, W. Theory of the flow of electrons and holes in germanium and other semiconductors. *Bell Syst. Tech. J.* **29**, 560-607 (1950).
3. Coldren, L. A., Corzine, S. W. & Mashanovitch, M. L. *Diode Lasers and Photonic Integrated Circuits*. (John Wiley & Sons, 2012).
4. Ferry, D. *Semiconductor Transport*. (Taylor & Francis, 2000).
5. Chazalviel, J. N. *Coulomb Screening by Mobile Charges: Applications to Materials Science, Chemistry, and Biology*. (Birkhäuser Boston, 1999).
6. Neamen, D. A. *Semiconductor Physics and Devices: Basic Principles*. (McGraw-Hill, 2012).
7. Klaassen, D. B. M. A unified mobility model for device simulation—I. Model equations and concentration dependence. *Solid-State Electronics* **35**, 953-959 (1992).
8. Caughey, D. M. & Thomas, R. E. Carrier mobilities in silicon empirically related to doping and field. *Proceedings of the IEEE* **55**, 2192-2193 (1967).
9. Grumstrup, E. M. *et al.* Ultrafast Carrier Dynamics in Individual Silicon Nanowires: Characterization of Diameter-Dependent Carrier Lifetime and Surface Recombination with Pump–Probe Microscopy. *J. Phys. Chem. C* **118**, 8634-8640 (2014).
10. Dziewior, J. & Schmid, W. Auger coefficients for highly doped and highly excited silicon. *Appl. Phys. Lett.* **31**, 346-348 (1977).
